# Non-specific effects of rabies vaccine on the incidence of common infectious disease episodes: study protocol for a randomized controlled trial

**DOI:** 10.1186/s13063-020-04467-z

**Published:** 2020-06-16

**Authors:** Darryn Knobel, Christianah Ibironke Odita, Anne Conan, Donna Barry, Marshalette Smith-Anthony, Juliet Battice, Shianne England, Bradford D. Gessner

**Affiliations:** 1 Ross University School of Veterinary Medicine, Basseterre, Saint Kitts and Nevis; 2 University of Pretoria, Pretoria, South Africa; 3 EpiVac Consulting Services, Anchorage, AK USA

**Keywords:** Rabies vaccine, Non-specific effects of vaccines, Influenza-like illness, Upper respiratory disease, Diarrhea, Fever

## Abstract

**Background:**

Vaccines may cause non-specific effects (NSEs) on morbidity and mortality through immune-mediated mechanisms that are not explained by the prevention of the targeted disease. Much of the evidence for NSEs comes from observational studies with a high risk of bias, and there is a clear need for new data from randomized controlled trials. Recently, it was proposed that rabies vaccine has protective NSEs in people and in animals. The aim of the proposed study is to determine whether rabies vaccine reduces the incidence rate of episodes of common infectious disease syndromes in a population of veterinary students on the island of St. Kitts.

**Methods:**

The trial design is a single-site, two-arm, parallel-group, participant-blinded, randomized, placebo-controlled, two-sided comparative study, with an internal pilot study for blinded sample size re-estimation. Allocation to study arm is by block randomization stratified by sex within cohort with a 1:1 allocation ratio. The primary study outcome is the number of new weekly episodes of common infectious diseases including respiratory, diarrheal and febrile illnesses. A vaccine immunogenicity ancillary study is planned.

**Discussion:**

Demonstration of a non-specific protective effect of rabies vaccine against unrelated respiratory, gastrointestinal and febrile illnesses would provide supportive evidence for the design of similar studies in children in populations with a high burden of these illnesses.

**Trial registration:**

ClinicalTrials.gov, ID: NCT03656198. Registered on 24 August 2018.

## Administrative information

Note: the numbers in curly brackets in this protocol refer to Standard Protocol Items: Recommendations for Interventional Trials (SPIRIT) Checklist item numbers. The order of the items has been modified to group similar items (see http://www.equator-network.org/reporting-guidelines/spirit-2013-statement-defining-standard-protocol-items-for-clinical-trials/).

**Table:** 

Title {1}	Non-specific effects of rabies vaccine on the incidence of common infectious disease episodes: study protocol for a randomized controlled trial
Trial registration {2a and 2b}.	ClinicalTrials.gov, ID: NCT03656198. Registered on 24 August 2018
Protocol version {3}	3 January 2020 (v4.1)
Funding {4}	The study is funded by RUSVM Intramural Grant 43001–2019. Vaccines (Rabivax-S) and diluent are provided by Serum Institute of India Pvt. Ltd. (SIIPL). The cost for immunogenicity testing of sera samples by RFFIT assay is covered by SIIPL
Author details {5a}	^1^Ross University School of Veterinary Medicine, Basseterre, St. Kitts ^2^University of Pretoria, Pretoria, South Africa ^3^EpiVac Consulting Services, Anchorage, AK, USA
Name and contact information for the trial sponsor {5b}	Ross University School of Veterinary Medicine (RUSVM), West Farm, Basseterre, St. Kitts
Role of sponsor {5c}	RUSVM is the sponsor-investigator. The protocol was shared with SIIPL. SIIPL input was limited to the vaccine safety and immunogenicity components of the protocol

## Introduction

### Background and rationale {6a}

Non-specific effects (NSEs) of vaccines – also known as heterologous effects [1] – are those immune-mediated effects of vaccines on morbidity and mortality that are not explained by the prevention of the targeted disease [2]. While NSEs may hypothetically be detrimental or beneficial, the majority of robust data support beneficial NSEs, as summarized by a World Health Organization (WHO) report [3]. Most recently, an immunological mechanism has been identified to explain the protective effect of measles vaccine in preventing all-cause mortality [4]. A systematic review [5] of the evidence for NSEs of Bacillus Calmette-Guérin (BCG), diphtheria tetanus pertussis (DTP) and measles-containing vaccines (MCV) on all-cause mortality in children under 5 years of age suggested different potential mechanisms and outcomes. Furthermore, much of the evidence came from observational studies with a high risk of bias [5] and, thus, the importance and implications of these studies remain controversial [6]. There is a clear need for new data from randomized controlled trials (RCTs), but there are logistic and ethical challenges to trials in children with relevant vaccines in high-mortality settings [6].

Recently, it was proposed that rabies vaccine (a non-live vaccine) has protective NSEs in people and in animals [7–9]. A recent RCT evaluated the RTS,S malaria vaccine using two parallel age-groups: 6 to 12 weeks and 5 to 17 months, with the former control population receiving a serogroup C meningococcal conjugate vaccine and the latter rabies vaccine. This trial found that among the older children, the RTS,S intervention group had an increased risk of all-cause meningitis compared to controls and a higher incidence of cerebral malaria despite clear evidence that RTS,S prevented malarial episodes [10]. Among several possible explanations, one is that rabies vaccine provides a protective benefit [7, 8]. In the age group for which rabies vaccine was used as a control, the control group had a 90% lower incidence of meningitis and approximately 50% lower incidence of cerebral malaria. In a population-based cohort study, rabies vaccine was shown to be associated with decreased all-cause mortality in free-roaming dogs in a high-mortality setting [9]. Compared to the unvaccinated group, all-cause mortality rates in the vaccine group were lower by 56%, 44% and 16% in young, adolescent and adult dogs, respectively. A review of the literature revealed older studies in mice, using live attenuated rabies vaccine, that provided protection against *Klebsiella pneumonia* sepsis [11] and mortality following intracerebral injection of a neurotropic strain of herpes virus [12] (42% and 26% reduction in mortality, respectively). Studies have shown that part of the rabies virus (the nucleoprotein, which is present in the vaccine) acts as a non-specific immunological enhancer [13]. Further RCTs are needed to test for the effect in people.

A non-specific protective effect of rabies vaccine would have implications for vaccine programs globally, but most acutely for the prevention of rabies in endemic areas. Although rabies vaccine is known to be a safe and effective vaccine [14], its routine use as pre-exposure prophylaxis in children is not recommended as it is not cost-effective in most situations, in which the incidence of exposure to rabies is relatively low [15]. A substantial non-specific protective effect against other infections would improve the economic argument for pre-exposure rabies vaccine prophylaxis, and potentially could accentuate existing vaccines (such as pneumococcal, meningococcal and *Haemophilus influenzae* type b conjugate vaccines) in preventing central nervous system infections such as acute bacterial meningitis.

### Objectives {7}

#### Primary hypothesis

Compared to an unvaccinated control group, administration of at least one dose of a three-dose course of rabies vaccine to previously-unvaccinated subjects leads to at least a 25% relative reduction in the rate of self-reported new episodes of common infectious disease syndromes (respiratory, diarrheal and febrile illness) over a 26-week period.

#### Primary objective

To determine whether the incidence rate of self-reported episodes of common infectious disease (CID) syndromes (respiratory, diarrheal and febrile illness) over a 26-week period is significantly different between previously unvaccinated subjects who receive at least one dose of a three-dose course of rabies vaccine and those subjects who receive a placebo injection. Primary analysis will be based on an intention-to-treat analysis.

#### Secondary objectives

- To compare, between the same two groups over the same time period:
  1. The rate of self-reported new episodes of respiratory illness (upper respiratory illness (URI) *or* influenza-like illness (ILI)), diarrhea (DIA) and undifferentiated febrile illness (UFI)
  2. The rates of self-reported new episodes of each syndrome separately:
    - ILI
    - URI
    - DIA
    - UFI
  3. The rate of clinically confirmed episodes of the study syndromes reported to RUSVM Health Services using the following the *International Classification of Diseases, version 10* (*ICD-10*) codes to define syndromes:
    - URI: J00 (acute nasopharyngitis)
    - ILI: J11 (influenza due to unidentified influenza virus)
    - DIA: R19.7 (diarrhea)
    - UFI: R50.9 (fever, unspecified)
  4. The rate of laboratory-confirmed episodes of respiratory illness (ILI or URI) and DIA
- To test for modification of effect of treatment on primary and secondary outcomes by sex

For secondary objective 1, in any week a single participant could report one of the following:

- No CID episode
- One CID episode (URI *or* ILI *or* DIA *or* UFI)
- Two CID episodes (either URI *or* ILI *and* DIA)

By this definition, a participant cannot experience more than two CID episodes within a week, as occurrence of URI together with ILI is considered an episode of ILI only, and occurrence of URI, ILI and/or DIA precludes the occurrence of UFI.

#### Safety objectives

- To compare, between the same two groups,
  1. The rate of solicited adverse events (AEs) through 3 days after each injection (dose 1, 2 and 3)
  2. The rate of unsolicited AEs and serious adverse events (SAEs) through 4 weeks after first injection
- To test for modification of effect of treatment on safety outcomes by sex

### Trial design {8}

The trial design is a single-site, two-arm, parallel-group, participant-blinded, randomized, placebo-controlled, two-sided comparative study, with an internal pilot study for blinded sample size re-estimation. Allocation to study arm is by block randomization stratified by sex within cohort (semester) with a 1:1 allocation ratio. An immunogenicity ancillary study is planned.

## Methods: participants, interventions and outcomes

### Study setting {9}

The study is taking place at Ross University School of Veterinary Medicine (RUSVM) on the island of St. Kitts in the Caribbean. The Doctor of Veterinary Medicine (DVM) program at RUSVM includes a preclinical curriculum of seven semesters in St. Kitts. There are three semesters per year (starting in January, May and September), and one intake (class) per semester. Each semester is 15 weeks, with a break of 2 or 3 weeks between semesters. RUSVM also offers a one-semester Veterinary Preparatory (VP) program. Students who successfully complete the VP program are placed into the first semester class. As St. Kitts is free of rabies, students do not need to be immunized during their preclinical training (semesters 1–7), but are required to receive the vaccine prior to attending clinical training off-island (semesters 8–10). The study enrolls participants from the VP program and the first and fifth semester classes of the DVM program each semester.

### Eligibility criteria {10}

#### Inclusion criteria

A student registered at RUSVM is eligible for inclusion in the study if they are in the VP program or the first or fifth semester of the DVM program.

#### Exclusion criteria

A student registered at RUSVM and in the VP program or the first or fifth semester of the DVM program is excluded from the study if they:

1. Have previously received a dose of rabies vaccine, or
2. Are intending to undertake activities during the course of participation in the study that would increase their risk category of rabies exposure above that of the US population at large, as defined by the Advisory Committee on Immunization Practices (ACIP) for human rabies prevention [16], or
3. Do not provide informed consent for participation, or
4. Enroll in the study but do not present for the first injection within the first 12 weeks of the semester (up to and including Week 12), or
5. Have a contraindication to rabies vaccine as described in the Rabivax-S package insert

### Who will take informed consent? {26a}

An invitation to join the study is sent by email to all students who meet the inclusion criteria. The email contains a unique link to a survey, which is used to establish eligibility (exclusion criteria) and seek agreement to participate from eligible students. Initial agreement to participate in the study is documented electronically as selection of the option, “I agree to participate in the study” for the unique survey linked to the participant’s identifiers (name, family name and email address). Participants who agree in the online survey still need to sign a paper copy of the informed consent document in person with a designated member of the study team (principal investigator (PI) or study coordinator). Only participants who sign a paper copy of the informed consent document with a designated member of the study team are considered enrolled and are allocated to a study group. A signed and dated copy of the consent form is given to the participant. Paper copies of signed consent forms are collected and stored by the PI in a locked file cabinet in an area with limited access (PI’s office). Following enrollment, participants are given a baseline health questionnaire to complete, and instructions to visit the RUSVM Health Services Office the following week (Monday through Friday) for screening and allocation into one of the two study arms. Enrollment and allocation of participants takes place within the first 2 weeks of each semester.

### Additional consent provisions for collection and use of participant data and biological specimens {26b}

Consent for collection and use of biological specimens (blood) in the vaccine immunogenicity ancillary study is included in the informed consent.

## Interventions

### Explanation for the choice of comparators {6b}

To maintain participant blinding, the Rabivax-S vaccine diluent (sterile water for injection) was selected as a comparator.

### Intervention description {11a}

The intervention for the treatment group is at least one dose (1 mL by intramuscular injection) of a three-dose primary course of Rabivax-S, on days 0, 7 and 21. Rabivax-S is a lyophilized vaccine manufactured by SIIPL containing inactivated purified rabies virus (Pitman Moore, PM3218 as virus strain) produced using Vero ATCC CCL 81 cells. The diluent (sterile water for injection) is provided in a separate 1-mL ampoule. After reconstitution, a single dose of 1 mL contains an inactivated, purified rabies antigen (not less than 2.5 IU), glycine (40 mg), sucrose (40 mg) and human serum albumin (25% 10 mg).

The intervention for the control group is at least one dose (1 mL by intramuscular injection) of a three-dose course of vaccine diluent (sterile water for injection), on days 0, 7 and 21.

### Criteria for discontinuing or modifying allocated interventions {11b}

Interventions will be discontinued in the event of a SAE or an AE categorized as severe and related to the study intervention (see the “Adverse event reporting and harms {22}” section).

### Strategies to improve adherence to interventions {11c}

Not applicable

### Relevant concomitant care permitted or prohibited during the trial {11d}

No restrictions are placed on concomitant care and interventions. Participants are screened at the time of presentation for first injection by study personnel at RUSVM Health Services, prior to assignment to study intervention. Study intervention is postponed for participants taking antimalarial medication or other short-term immunosuppressive treatments.

### Provisions for post-trial care {30}

Ross University School of Veterinary Medicine has insurance to cover for wrongful acts arising out of the rendering/failure to render professional services (including those associated with the protocol). This will include cover for additional health care, compensation or damages awarded by claims pursued through the courts. Incidences judged to arise from fraudulent, criminal or intentional acts (including those due to major protocol violations) will not be covered by study insurance policies. The liability of the manufacturer of Rabivax-S (SIIPL) is strictly limited to those claims arising from faulty manufacturing of the product and not to any aspects of the conduct of the study.

### Outcomes {12}

#### Primary outcome measure

The incidence rate of self-reported, new, weekly episodes of acute common infectious disease (CID), defined as any of the following: URI *or* ILI *or* DIA *or* UFI.

URI is defined as (two or more of the following: runny or blocked nose/sneezing/sore throat/cough) and (absence of itchy or watery eyes).

ILI is defined as fever (feeling feverish, or an axillary, oral or otic temperature of 100 °F or higher) and (cough or sore throat).

DIA is defined as three or more loose stools within a 24-h period

UFI is defined as fever (feeling feverish, or an axillary, oral or otic temperature of 100 °F or higher) and (not meeting the case definition of URI, ILI or DIA).

Participants provide weekly self-reports of occurrence or non-occurrence of episodes of CID per week for a maximum of 26 weeks, starting 10–14 days after allocation. To be defined as a new episode, illness must be preceded by at least 1 week in which no CID episode is reported.

#### Secondary outcome measures

1. Incidence rate of self-reported new episodes of respiratory illness (URI or ILI), DIA and UFI
2. Incidence rate of self-reported new weekly episodes of URI
3. Incidence rate of self-reported new weekly episodes of ILI
4. Incidence rate of self-reported new weekly episodes of DIA
5. Incidence rate of self-reported new weekly episodes of UFI, and
6. Incidence rate of clinically confirmed episodes of CID syndromes. Clinically confirmed episodes of CID syndromes are defined as an episode resulting in a visit to the RUSVM Health Services with a recorded *ICD-10* of J00 (acute nasopharyngitis); J11 (influenza due to unidentified influenza virus); R19.7 (diarrhea) or R50.9 (fever, unspecified)
7. Incidence rate of laboratory-confirmed episodes of respiratory illness (URI or ILI) or DIA, defined as clinically confirmed episodes with laboratory diagnosis of influenza virus, respiratory syncytial virus or metapneumovirus (URI/ILI episodes) or rotavirus or norovirus (DIA episodes)

#### Safety outcome measures

1. Number of solicited self-reported events over 3 days following each injection of the following AEs:
  - Local reactions (limited to the site of the injection): pain, erythema, edema, pruritus and induration
  - Systemic reactions: fever, shivering, malaise, asthenia, faintness, dizziness, headache, myalgia, arthralgia, nausea and abdominal pain
  - Hypersensitivity or allergic reactions: anaphylaxis, urticaria, rash and erythema multiforme
2. Number of unsolicited AEs and SAEs reported to RUSVM Student Health Services through 4 weeks after first injection

#### Immunogenicity ancillary study outcome measures

Geometric mean concentration (GMC) of rabies-virus neutralizing antibody (RVNA) titers in IU/mL measured by rapid fluorescent foci inhibition test (RFFIT) on sera samples collected 3 to 11 days before injection (pre-injection) and 173 to 187 days after first injection (post-injection). Proportion of participants with RVNA titers ≥ 0.5 IU/ml post-injection (WHO recommendation). Proportion of participants with RVNA titers ≥ 0.1 IU/ml post-injection (ACIP recommendation).

### Participant timeline {13}

**Fig. 1:**
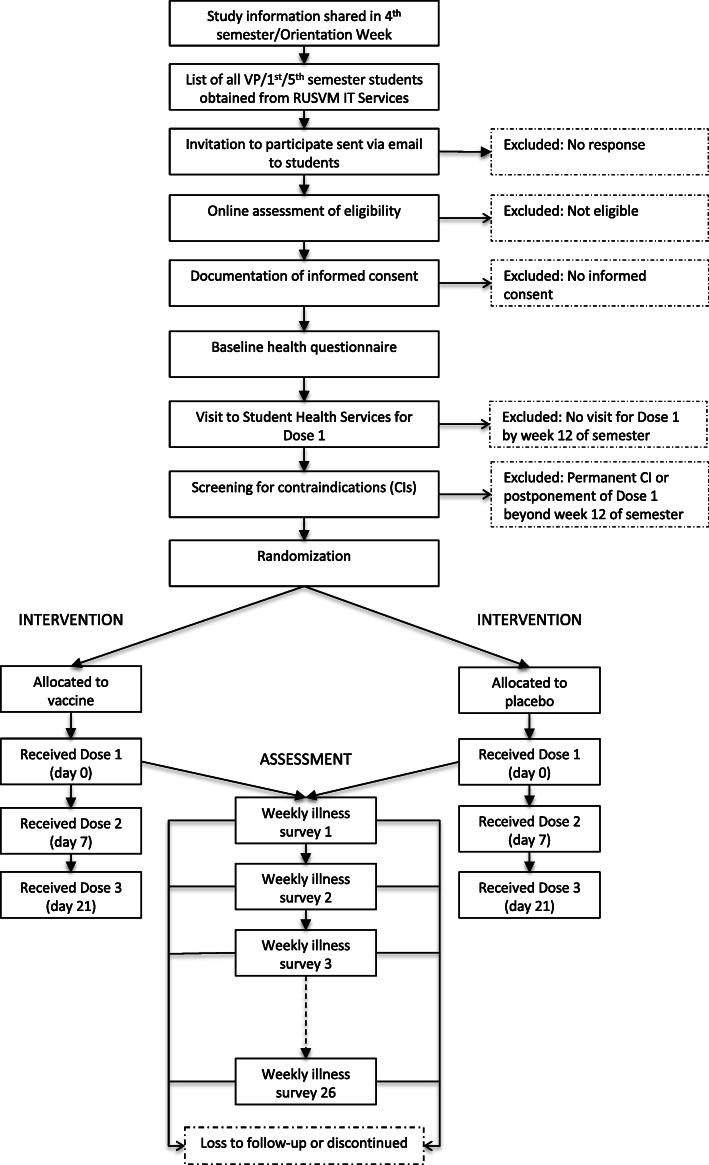
Participant flow diagram

The participant flow diagram is shown in Fig. 1 and the schedule of activities is shown in Table 1.

**Table 1:** Schedule of activities for participants in a single cohort (semester)

		1st semester						Semester break^a^		2nd semester					
Weeks since allocation (study week)		Wk -1	Wk 1	Wk 2	Wk 3	Wk 4	Etc.	Wk 15	Wk 16	Wk 17	Etc.	Wk 25	Wk 26	Wk 27	Wk 28
*Weeks of semester/break*		*Wk 1*	*Wk 2*	*Wk 3*	*Wk 4*	*Wk 5*	*Etc.*	*Wk 1*	*Wk 2*	*Wk 1*	*Etc.*	*Wk 8*	*Wk 9*	*Wk 10*	*Wk 11*
Enrollment															
Information	X	X													
Eligibility screen	X	X													
Informed consent	X	X													
Contraindication screening			X												
Allocation			X												
Intervention^b^															
Dose 1			X												
Dose 2				X											
Dose 3						X									
Assessments															
Baseline		X													
Weekly illness survey (1–26)					X (S1)	X (S2)	X (Etc.)	X (S 13)	X (S 14)	X (S 15)	X (Etc.)	X (S 23)	X (S 24)	X (S 25)	X (S 26)
Solicited adverse events			X	X		X									
Unsolicited adverse events			X	X	X	X									
Blood collection (efficacy ancillary study)		X (D -7)										X (D 180)			
Blood collection (immunology ancillary study)			X (D 0)			X (D 21)		X (D 90)							
Study exit															X

^a^Some semester breaks are 3 weeks long, in which case Weeks 27 and 28 since allocation will fall in Weeks 9 and 10 of the sixth semester

^b^Schedule shown here assumes administration of the first dose in Week 2 and administration of subsequent doses per schedule (day 7 and day 21). If necessary, participants can begin the intervention at any time in the first 12 weeks of the semester

### Sample size {14}

The estimated number of participants needed to achieve the study’s primary objective is 430 (215 in each group). The sample size was calculated using the approach for comparing two negative binomial rates based on true rates (Approach 2 in [17]), implemented using the function *power.nb.test* in package “MKmisc” [18] in R software [19], and assuming the following parameter values:

- Alpha level = 0.05
- Targeted power = 0.8
- Event rate for control group = 2 (expected mean number of new CID episodes over 26 weeks)
- Rate ratio under alternative hypothesis = 0.75
- Average length of participation (accounting for drop-out and non-response) = 21 weeks (0.8 of 26-week observation period)
- Negative binomial dispersion parameter (*k* in [18]) = 0.4

The effect size (rate ratio 0.75, or 25% relative reduction) is based on the range of effect sizes seen for other outcomes in people and in animal studies [7–12]. The three nuisance parameters (average length of participation, event rate for control group and negative binomial dispersion parameter) were estimated from data collected over 7 weeks of a pilot study of rates of CID episodes in 90 RUSVM students (40 in the first semester and 50 in the fifth semester) from 21 May to 8 July 2018 (Weeks 3 through 9 of the summer semester).

An internal pilot study will be done using data from the first 300 participants to complete the study. As the expected enrollment rate is < 100 participants per semester, this will allow capture of seasonal variation in event rates (three or more semesters in an internal pilot study). The three nuisance parameters will be re-estimated from the internal pilot study data and the sample size recalculated, using a blinded sample size re-estimation method for count data [20, 21], which maintains required power without an increase in the type I error. The sample size will only be increased and not decreased on the basis of the internal pilot study (that is, if the recalculated sample size is smaller than the original sample size, the original sample size will still be used).

### Recruitment {15}

The PI or study coordinator presents information on the study to prospective participants prior to enrollment. Prospective participants are provided with a link to a website that contains more information about the study, including the informed consent documents and contact details of the study personnel. A question-and-answer session with the PI is scheduled. Prospective participants have the opportunity to ask questions via email or in person with the study investigators, either during scheduled information sessions or by appointment. To compensate study participants for their time and inconvenience, the study provides the primary course of vaccine at no cost to all participants, including participants in the control group following exit from the study (normal cost through RUSVM Health Services is US$200).

## Assignment of interventions: allocation

### Sequence generation {16a}

Allocation of participants to study arms is done by restricted randomization (permuted block design with stratification). Stratification is by cohort (three cohorts per year: January, May and September) and sex (within cohort). Within strata, randomization is done by computer-generated randomly permuted blocks of varying size, using the function *blockrand* in the package “blockrand” [22] in R software [19]. Enrolled participants are allocated in a 1:1 ratio to one of the two study arms.

### Concealment mechanism {16b}

Randomization cards are created by the PI or the study coordinator (using the function *plotblockrand* in the package blockrand [22] in R), placed in opaque envelopes with a window, and sealed, with the sequential subject number (starting from 1 for each sex in each cohort) visible in the envelope window. Envelopes are drawn sequentially by study personnel at RUSVM Health Services as participants come in for the first injection (day 0), and opened to determine allocation. After the injection is administered, the RUSVM Health Services member completes the visit case report form (CRF), including participant name, surname, email address, allocation group, intervention actually received, date received, and the batch number and expiry date of the vaccine or diluent. An entry is also made in students’ health records. Data from the visit CRF are entered into a password-protected file in a restricted-access folder on the RUSVM network. Access to the folder is restricted to study personnel only. When participants return for the second (day 7) and third (day 21) injections in the course, study staff at RUSVM Health Services refer to the file to determine the participant’s allocation.

### Implementation {16c}

Allocation sequence is generated by the PI. Enrollment of participants is by the PI and study coordinator. Assignment of participants to interventions is by the study nurses.

## Assignment of interventions: blinding

### Who will be blinded? {17a}

Participants are blinded to their study arm allocation. The intervention procedure is identical for both arms (intramuscular injections at RUSVM Health Services on days 0, 7 and 21). Participants allocated to the control group receive an intramuscular injection of sterile water using identical syringes and needles as for the vaccine group. The injection is prepared in a separate room to maintain participant blinding.

Analytical blinding will be maintained until completion of the blinded sample size re-estimation. This is done by maintaining intervention assignment and outcome measures in separate datasets. Thereafter, analytical blinding will be maintained for each study cohort until exit of that cohort, whereupon the intervention assignment and outcome measures datasets will be merged for analysis.

### Procedure for unblinding if needed {17b}

Unblinding of participants occurs when participants exit the study or in circumstances in which there is a need to determine their rabies pre-exposure vaccination status; for example, in the event of a possible rabies exposure or if participants wish to undertake activities during the course of their participation in the study that would increase their risk category of rabies exposure. Rabies post-exposure management differs between patients who have not been previously vaccinated and those who have been previously vaccinated. Thus, in the event of a suspected rabies exposure that requires post-exposure prophylaxis, participants should establish their vaccination status as soon as possible. Steps for determining vaccination status and emergency contact details of all relevant personnel are provided in the informed consent document and in the weekly survey sent to participants. Participants who are unblinded exit the study at that point.

## Data collection and management

### Plans for assessment and collection of outcomes {18a}

After enrollment, participants complete a questionnaire that captures data on relevant baseline characteristics including sex, age, health and vaccination status. Participants complete a short survey each week, starting in study Week 3, to capture self-reported episodes of illness (respiratory, diarrheal and febrile illness; primary outcome measure and secondary outcome measures 1–5) in the preceding week. Surveys are sent by email to all participants each Monday. Participants who have not completed the survey by Wednesday afternoon are sent a request by email to complete the survey for the preceding week. Surveys are sent weekly, for 26 weeks. A participant is considered to have completed the study 1 week after receipt of the final (26th) weekly survey for that participant’s cohort.

Clinically confirmed episodes of study syndromes (secondary outcome measure 6) are collected by study personnel at RUSVM Health Services, as part of the routine clinic visits and diagnostics. At the end of each 26-week observation period for each study cohort, data from that cohort are compiled by the manager of the RUSVM Health Services, anonymized to protect privacy and aggregated by study arm, before being sent to the data analyst who will conduct the analysis.

Occurrence of expected AEs is solicited in an online survey sent to participants 3 days after receiving an injection (for doses 1, 2 and 3). Occurrences of unsolicited AEs and SAEs are captured by study personnel at RUSVM Health Services.

### Plans to promote participant retention and complete follow-up {18b}

Each week that a participant submits a weekly illness survey, they stand a chance to win a EC$25 gift voucher to a food/beverage vendor of their choice on the RUSVM campus. Each week’s winner is picked at random from the names of all participants who complete a survey that week.

A participant is considered lost to follow-up if they fail to complete a weekly survey for more than three consecutive weeks and is unable to be contacted by the study personnel. Contact is by email to the participant’s registered RUSVM email address. The participant is considered unreachable if they do not respond after 3 emails. At this point, they are considered to have withdrawn from the study with a primary reason of lost to follow-up.

### Data management {19}

Information from the baseline health questionnaire and study CRFs are entered in EpiInfo™ by the study research assistant. Data entry is checked by the PI against the original hard copy. Solicited AEs and weekly survey data on illness episodes are collected through the Qualtrics® platform. Survey data are accessible only by study investigators. Weekly survey data are downloaded by the data analyst from Qualtrics servers each week. Downloaded data are stored on a password-protected computer and backed up to an encrypted external hard drive.

For the vaccine immunogenicity ancillary study, RFFIT RVNA titers are sent by the laboratory to the PI or study coordinator, who enters the data in the CRF.

### Confidentiality {27}

All study-related information is stored electronically in password-protected databases in file folders with restricted access on the RUSVM network or on password-protected laptops with back-up to encrypted external hard drives. Any paper copies with participant information is stored in locked file cabinets in areas with limited access. Biological specimens are identified by a coded identification number. All records that contain names or other personal identifiers are stored separately from the code list.

### Plans for collection, laboratory evaluation and storage of biological specimens for genetic or molecular analysis in this trial/future use {33}

Participants in the vaccine immunogenicity ancillary study have a maximum volume of 8 mL blood collected by venipuncture prior to injection on day − 7 (acceptable range − 3 through − 11), and again on day 180 (acceptable range 166 through 194). Blood collection is done by external service providers (Avalon Laboratories). Serum is separated into two aliquots. One aliquot is sent by Avalon Laboratories to the Rabies Laboratory at Kansas State University for measurement of RVNA by RFFIT. The second aliquot is retained and stored at − 20 °C at RUSVM as back-up in case of loss of the first aliquot. All unused material, including the second aliquot, will be destroyed at the end of the study.

For secondary outcome measure 7 (incidence rate of laboratory-confirmed episodes of respiratory illness (URI or ILI) or DIA), etiological confirmation will be done for a limited range of pathogens only (influenza virus, respiratory syncytial virus, metapneumovirus, rotavirus and norovirus) through an independent research protocol (RUSVM Institutional Review Board (IRB) protocol #17–11-FL) and the test results sent back to the RUSVM Health Services. Data are compiled by the manager of the RUSVM Health Services, anonymized to protect privacy and aggregated by study arm, before being sent to the Data Analyst who will conduct the analysis.

## Statistical methods

### Statistical methods for primary and secondary outcomes {20a}

The analysis of the primary and secondary outcomes 1–7 will be conducted after all randomized participants have completed the study (1 week after receipt of the final weekly survey) or have withdrawn from the study, whichever occurs first. Primary analysis will be based on an intention-to-treat analysis of all events (that is, not just occurrence of first events). The comparison of incidence rates of CID episodes (primary outcome and secondary outcomes 1–5) between the treatment arms will be performed using a negative binomial regression model, which accounts for different lengths of observation period. Participants’ number of CID episodes will be modeled as a function of treatment group, with the number of weeks of observation included as an offset in the model. The model will also include sex and cohort, as stratification factors in the randomization. This analytic model estimates the rate ratio, λ_t_/ λ_c_, which quantifies the relative difference in rates of CID episodes associated with rabies vaccination (λ_t_) in comparison to placebo injection (λ_c_). A rate ratio of 1 would be consistent with no effect of treatment. Statistical significance will be controlled at the two-sided, 0.05 alpha level, and the estimated rate ratio compared with 1, assuming the following statistical hypothesis:

- *H*_*0*_ (null hypothesis): rate ratio = 1
- *H*_*1*_ (alternative hypothesis): rate ratio ≠ 1

Statistical significance at the pre-specified alpha level will be based on a Wald testing procedure. CID rates (primary endpoint and secondary endpoints 1–6) for rabies vaccine and placebo, and the rate ratio, will be presented and will include 95% confidence intervals.

Outcomes 6 (number of clinically confirmed episodes recorded at RUSVM Health Services) and 7 (number of laboratory-confirmed episodes) will be available in aggregate for each treatment group and cohort. We will use a Poisson or negative binomial regression model with treatment and cohort as group-level covariates and group size as offset. The choice of regression model will be based on the likelihood ratio test, comparing the fitted Poisson to the fitted negative binomial model.

Immunogenicity ancillary study outcome measures will be compared between treatment groups using two-sided tests (Wilcoxon rank sum test for RNVA titers, and Fisher’s exact test for proportions).

### Interim analyses {21b}

No interim analyses will be conducted.

### Methods for additional analyses (e.g., subgroup analyses) {20b}

Analyses for modification of effects by sex, season (spring = January cohorts; summer = May cohorts; winter = September cohorts) and new (less than one semester on the island) vs. old (more than four semesters on the island) arrivals will be done by a formal statistical test for interaction (likelihood ratio test) at the 0.05 alpha level, by inclusion of the interaction term between treatment and sex in the negative binomial model.

Subset analyses of the primary outcome measure and secondary outcome measures 1–7 will be conducted in the subset of participants excluding those participants categorized as immunosuppressed (reporting use of immunosuppressive medications or treatments or occurrence of immunosuppressive medical conditions in baseline questionnaire).

### Methods in analysis to handle protocol non-adherence and any statistical methods to handle missing data {20c}

Primary analysis will be based on an intention-to-treat analysis (as randomized). Secondary analyses will include per-protocol analysis (receipt of all three doses) and extended per-protocol analysis (receipt of at least one dose) of primary and secondary outcomes.

We intend to conduct a complete case analysis for treatment allocation, primary and secondary outcomes, and main potential effect modifier (sex), although we will inspect patterns of missing data and may consider a method such as multiple imputation. Missing data on other covariates for secondary or tertiary analyses will be handled through multiple imputation.

### Plans to give access to the full protocol, participant-level data and statistical code {31c}

A deidentified participant-level dataset and statistical code will be provided by the investigators upon request.

## Oversight and monitoring

### Composition of the Coordinating Center and Trial Steering Committee {5d}

Not applicable.

### Composition of the Data Monitoring Committee (DMC), its role and reporting structure {21a}

A DMC was not warranted as the treatment intervention is a licensed vaccine pre-qualified by the WHO and with known minimal risks.

### Adverse event reporting and harms {22}

#### Definitions of adverse events and serious adverse events

Definitions of AEs and SAEs are taken from the Office of Human Research Protections Guidance on Reviewing and Reporting Unanticipated Problems Involving Risks to Subjects or Others and Adverse Events.

Adverse event means any untoward or unfavorable medical occurrence in a participant, including any abnormal sign (for example, abnormal physical exam or laboratory finding), symptom or disease, temporally associated with the subject’s participation in the research, whether or not considered related to the subject’s participation in the research.

A SAE is any AE temporally associated with the subject’s participation in research that meets any of the following criteria:

1. Results in death
2. Is life-threatening (places the subject at immediate risk of death from the event as it occurred)
3. Requires inpatient hospitalization or prolongation of existing hospitalization
4. Results in a persistent or significant disability/incapacity
5. Results in a congenital anomaly/birth defect; or
6. Any other AE that, based upon appropriate medical judgment, may jeopardize the subject’s health and may require medical or surgical intervention to prevent one of the other outcomes listed in this definition (examples of such events include allergic bronchospasm requiring intensive treatment in the emergency room or at home, blood dyscrasias or convulsions that do not result in inpatient hospitalization, or the development of drug dependency or drug abuse)

#### Classification of adverse events

All AEs will have their relationship to study intervention assessed by the study nurse practitioner), in consultation with the study medical advisor, based on temporal relationship and clinical judgment.

The following guidelines will be used to describe severity:

- *Mild* – Events require minimal or no treatment and do not interfere with the participant’s daily activities
- *Moderate* – Events result in a low level of inconvenience or concern with the therapeutic measures. Moderate events may cause some interference with functioning
- *Severe* – Events interrupt a participant’s usual daily activity and may require systemic drug therapy or other treatment. Severe events are usually potentially life-threatening or incapacitating. Of note, the term “severe” does not necessarily equate to “serious”

The degree of certainty about causality will be graded using the categories below:

- *Related* – The AE is known to occur with the study intervention, there is a reasonable possibility that the study intervention caused the AE, or there is a temporal relationship between the study intervention and event. Reasonable possibility means that there is evidence to suggest a causal relationship between the study intervention and the AE
- *Not related* – There is not a reasonable possibility that the administration of the study intervention caused the event, there is no temporal relationship between the study intervention and event onset, or an alternate etiology has been established

Adverse events will also be classified as *expected* or *unexpected*. An AE will be considered unexpected if the nature, severity or frequency of the event is not consistent with the risk information previously described for the study intervention. Based on the information contained in the package insert for Rabivax-S, the following AEs are considered expected:

- Local reactions (limited to the site of the injection): pain, erythema, edema, pruritus and induration
- Systemic reactions: fever, shivering, malaise, asthenia, faintness, dizziness, headache, myalgia, arthralgia, nausea and abdominal pain
- Hypersensitivity or allergic reactions: anaphylaxis, urticaria, rash and erythema multiforme

#### Identification of adverse events

The Study Coordinator will review the visit 1–3 CRFs daily for participants receiving injections (doses 1, 2 or 3). The study coordinator will send a link to an online Qualtrics survey by email on day 3 after the date of the injection (doses 1, 2 or 3) to all participants. Information on occurrence of expected AEs will be specifically solicited in the survey, by asking the question, “Since receiving the study injection 3 days ago, did you experience any of the following…?” and providing participants with a list of expected AEs (see the “Classification of adverse events” section for details) from which to select. Participants will also be asked to grade the severity of any AEs (mild, moderate or severe) and the outcome (recovered/resolved without sequelae, recovered/resolved with sequelae or ongoing). Information on occurrence of other AEs (unsolicited) will be sought by asking the question, “Besides the signs listed above, have you noticed anything different since starting the study?” and providing participants the opportunity to describe what they noticed or experienced, and grade the severity and outcome as above.

Participants will also be encouraged to report any AEs to RUSVM Health Services (unsolicited AEs). An AE form will be completed for all AEs reported to RUSVM Student Health Services, using the Vaccine Adverse Event Reporting System (VAERS) writable PDF form (https://vaers.hhs.gov/pdf/vaers_form.pdf). The PI, together with the manager of the RUSVM Student Health Services, will review and compare the reports of unsolicited AEs (AE forms) against reports of solicited AEs in the AE surveys, to avoid double capture.

#### Reporting of adverse events

The PI will report all SAEs whether or not considered study intervention related, and all non-serious unexpected AEs considered related to the study intervention (see the “Classification of adverse events” section for details), to the RUSVM IRB within 48 h of his becoming aware of the event (through weekly review, or through notification from study personnel at the RUSVM Student Health Services).

The PI will report safety data (AEs and SAEs) to the vaccine manufacturer for the first two cohorts within 30 days of 80% of the cohort completing 4 weeks’ follow-up after first dose. For remaining cohorts, RUSVM will report safety data to the vaccine manufacturer for each cohort within 30 days of exit of participants from that cohort.

### Frequency and plans for auditing trial conduct {23}

Trial conduct is audited by the RUSVM Institutional Officer through observation of participant enrollment, allocation and intervention, and inspection of trial records.

### Plans for communicating important protocol amendments to relevant parties (e.g., trial participants, ethical committees) {25}

Any modifications to the protocol that may impact on the conduct of the study, potential benefit of the patient or may affect patient safety, including changes of study objectives, study design, patient population, sample sizes, study procedures or significant administrative aspects will require a formal amendment to the protocol. Such amendments will be submitted to the RUSVM IRB for review and approval prior to implementation.

Administrative changes of the protocol are minor corrections and/or clarifications that have no effect on the way the study is to be conducted. These administrative changes will be documented in a memorandum. During annual continuing review of the study, the RUSVM IRB will be notified of administrative changes made to the protocol since the previous review.

### Dissemination plans {31a}

Trial results will be disseminated by publication in a scientific journal and by updating the trial registry. Results will also be included in a PhD thesis by the PhD student. Results will be disseminated regardless of the magnitude or direction of effect. Communication of findings to a non-academic audience will be through the RUSVM website, social media, a press release from RUSVM communications, and articles for non-academic audiences in the popular press.

## Discussion

This paper describes the protocol for a RCT of the non-specific effect of rabies vaccine on the incidence of common infectious disease episodes in a population of veterinary students. Gessner et al. [7] proposed that rabies vaccine may have a non-specific protective effect, as an alternative explanation for the higher rates of central nervous system infections seen in the treatment arm of the RTS,S malaria vaccine study compared to the control arm. Examination of this proposition in studies such as ours may help to distinguish between these two explanations. If our study demonstrates a beneficial non-specific effect, it would be the first to do so for a non-live vaccine.

Some practical and operational issues were encountered in performing the study. Secondary outcome 7 (laboratory confirmation of episodes of respiratory illness and diarrhea) was dropped and this objective abandoned due to low sample submission numbers under the independent research protocol related to that objective.

Due to AEs related to the intramuscular injection of sterile water, the placebo was changed to sterile saline for injection on 13 September 2019, during allocation of the fourth cohort.

The blinded sample size re-estimation for the primary study objective was conducted on 22 November 2019, based on data from the first three cohorts to complete the study (*n* = 351). The recalculated sample size is 584. The values of the three nuisance parameters based on this re-estimation were: average length of participation 21 weeks; event rate for control group (under alternative hypothesis) 1.3; negative binomial dispersion parameter 0.5. Thus, the increase in sample size is largely due to the lower-than-expected rate of new episodes of CID. This may be due to the definition of a new episode, in which illness must be preceded by at least 1 week in which no CID episode is reported. Illness events which were reported in the first week of the survey or which were otherwise preceded by a non-response of one or more weeks were not included as new episodes. This definition was not applied in the pilot study, which may account to some extent for the lower event rate in the main study.

The summer 2020 semester was moved online due to travel and health restrictions related to the Covid-19 pandemic; thus, enrollment of the final cohort is postponed until on-campus teaching resumes. The post-vaccination blood draw of the Fall 2019 cohort was postponed for the same reason.

Our study protocol has several limitations. Allocation concealment using opaque, sequentially numbered envelopes is susceptible to subversion, but technological limitations and the rapid pace of allocation (as participants would arrive in groups on their given day) precluded the use of more sophisticated methods. Adequacy of randomization and allocation concealment will be checked through comparison of baseline variables between the treatment groups. Our study relies on self-reported episodes of illness by participants. Blinding participants to their treatment group allocation will reduce the risk of misclassification bias inherent in a subjective outcome measure. To reduce risk of selection bias due to non-response and drop-out, we have purposefully kept the weekly illness survey brief. A resulting limitation of our study is a lack of information on disease severity. It is plausible that a non-specific effect could affect severity but not incidence of infection; such an effect would not be detected in our study. Although we have selected syndromes that would result from common infections, we cannot distinguish cases that arise from non-infectious processes.

Future trials of non-specific effects of vaccines will benefit from examination of the biological mechanisms through which any effects are exerted. Techniques of mediation analysis in a causal inference framework, which would allow decomposition of the effect of a vaccine through different biological pathways, could provide a valuable tool for these endeavors [23].

## Trial status

Enrollment began on 29 August 2018. The trial is currently enrolling participants by invitation. Enrollment is anticipated to be completed by September 2020. The protocol ID is 18–04-FL version 4.1, dated 3 January 2020.

## Data Availability

Data sharing is not applicable to this article as no datasets were generated or analyzed. For the trial, a deidentified participant-level dataset and statistical code will be provided by the investigators upon request.

## Abbreviations

ACIP: Advisory Committee on Immunization Practices
AE: Adverse event
BCG: Bacillus Calmette-Guérin
CID: Common infectious disease
DIA: Diarrhea
DTP: Diphtheria tetanus pertussis
DVM: Doctor of Veterinary Medicine
GMC: Geometric mean concentration
ILI: Influenza-like illness
IRB: Institutional Review Board
MCV: Measles-containing vaccines
NSE: Non-specific effect
RCT: Randomized controlled trial
RFFIT: Rapid fluorescent focus inhibition test
RUSVM: Ross University School of Veterinary Medicine
RVNA: Rabies-virus neutralizing antibodies
SAE: Serious adverse event
SIIPL: Serum Institute of India Pvt. Ltd.
UFI: Undifferentiated febrile illness
URI: Upper respiratory illness
VAERS: Vaccine Adverse Event Reporting System
VP: Veterinary Preparatory
WHO: World Health Organization
